# Hyponatremia as a Predictor of Complicated Appendicitis

**DOI:** 10.7759/cureus.96113

**Published:** 2025-11-04

**Authors:** Bandar AlMutairi, Ahmed M Alshammari, Lubna Alsuraykh, Norah H Alhumaidi, Arina M Alhamed, Rasha S Alhuwayri, Edward Mugambi Ireri

**Affiliations:** 1 General Surgery, Buraidah Central Hospital, Buraidah, SAU; 2 General Surgery, King Saud Medical City, Riyadh, SAU; 3 Medicine and Surgery, Unaizah College of Medicine and Medical Sciences, Unaizah, SAU; 4 Medicine and Surgery, College of Medicine, Qassim University, Buraidah, SAU; 5 Data Science and Analytics, Smart Health EQUAS Consultants Limited Company, Nairobi, KEN

**Keywords:** complicated acute appendicitis, hyponatremia, preoperative complications, risk group stratification, saudi arabia, serum sodium

## Abstract

Background

Acute appendicitis is one of the most common surgical emergencies, presenting as either uncomplicated or complicated disease. Complicated cases, characterized by perforation, abscess, or inflammatory mass, are associated with poorer outcomes and require timely identification to guide optimal management. Although several international studies have suggested that serum hyponatremia may predict complicated appendicitis, data from Middle Eastern populations remain limited.

Method

A quantitative, retrospective cohort study was conducted at Buraidah Central Hospital, Qassim Region, Saudi Arabia, to evaluate hyponatremia as a predictor of complicated acute appendicitis. Medical records of 965 patients diagnosed between January 2020 and December 2023 were reviewed using a census sampling design. Patients aged 14 years and above with complete records were included, while those with uncomplicated appendicitis, missing data, or comorbidities affecting sodium balance were excluded. Data on demographic, laboratory, and clinical variables were analyzed using Python. Inverse probability of treatment weighting was applied to minimize confounding, and statistical significance was set at p < 0.05.

Results

The overall prevalence of hyponatremia in the study population was 11.3%, and that among patients with complicated appendicitis was 4%. Hyponatremia occurred in 6.5% of pediatric and 11.8% of adult patients. In the gender-stratified model, age (adjusted OR [AdjOR] = 1.027; 95% CI: 1.001-1.053) and serum sodium (AdjOR = 1.207; 95% CI: 1.053-1.383) showed significant positive associations with complicated appendicitis. Similarly, in the model stratified by surgical procedure, age (AdjOR = 1.029; 95% CI: 1.004-1.055) and serum sodium (AdjOR = 1.168; 95% CI: 1.024-1.333) remained significant predictors. Contrary to the initial hypothesis and existing literature, higher serum sodium levels, rather than hyponatremia, were associated with an increased likelihood of complicated appendicitis.

Conclusion

This study highlights a paradoxical positive association between serum sodium levels and complicated appendicitis, differing from prior evidence that linked hyponatremia to severe disease. While serum sodium alone is not a reliable standalone predictor, its diagnostic value is enhanced when interpreted alongside other clinical and laboratory parameters. The combined assessment of sodium levels and patient age may improve early risk stratification and guide timely clinical decision-making.

## Introduction

Acute appendicitis is one of the most common surgical emergencies, with a lifetime prevalence of approximately 7-8% [1,2]. Patients may present with uncomplicated appendicitis, typically managed by appendectomy, or with diffuse peritonitis requiring urgent surgery. A subset of patients, however, presents with complicated appendicitis, characterized by a contained perforation, abscess, or inflammatory mass [3]. These patients generally experience worse outcomes compared to those with uncomplicated disease [4]. Early preoperative identification of complicated appendicitis is therefore essential to guide the timing of intervention and optimize treatment strategies [5].

Diagnosis of acute appendicitis relies on clinical evaluation, laboratory testing, and imaging [1]. Various laboratory parameters, imaging modalities, and clinical scoring systems have been proposed to improve diagnostic accuracy [6-9]. Among these, the Adult Appendicitis Score, the Appendicitis Inflammatory Response (AIR) score, and the RIPASA score demonstrate superior diagnostic performance compared with the Modified Alvarado Score [10]. However, each method has limitations, highlighting the need for additional, reliable biochemical markers [11].

Recent studies have investigated serum sodium (Na) (hyponatremia) as a potential predictor of complicated appendicitis because it is inexpensive, routinely measured, and widely available [11]. However, reported diagnostic accuracy varies considerably across populations. For instance, studies from Brazil and Turkey reported sensitivities of 45.7% and 63%, respectively, with specificities ranging from 66% to 86% [11,12]. Research in Kuwait found higher sensitivity (84.4%) but lower specificity (45.6%) [13]. A multicenter study in the United Kingdom observed inconsistent results between adults and children [14], while studies from Europe and South Asia similarly showed conflicting associations between serum Na and disease severity [15,16]. A meta-analysis of seven studies confirmed that children with complicated appendicitis tend to have lower serum Na levels than those with uncomplicated cases (p < 0.00001) [17].

Despite growing evidence, regional data from the Middle East remain scarce. Given the biological and population-level variability in hyponatremia, its predictive role warrants local validation.

Therefore, this study aimed to evaluate the predictive role of hyponatremia in complicated appendicitis among patients in Saudi Arabia, thereby providing population-specific evidence to inform early diagnosis and management. A secondary objective was to contextualize these findings within global literature and identify preoperative (demographic and clinical) as well as postoperative (complication-related) factors associated with complicated appendicitis. Specifically, the study sought to assess the association between serum Na levels (hyponatremia) and the presence of complicated appendicitis, identify clinical and procedural factors associated with complicated disease, and determine key demographic and biochemical predictors that may support early risk stratification and clinical decision-making.

## Materials and methods

Study design and setting

A quantitative, observational, analytical, retrospective cohort study design was used, with hyponatremia as a predictor of complicated acute appendicitis. The study was conducted at Buraidah Central Hospital, Qassim Region, in the Kingdom of Saudi Arabia.

Sample size and sampling technique

The study focused on medical records of complicated appendicitis from January 2020 to December 2023, reviewing a total of 965 patient records. A census sampling design was employed, capturing all consecutive patients diagnosed with complicated appendicitis who met the inclusion criteria during the study period.

Inclusion and exclusion criteria

The inclusion criteria comprised patients of both genders, aged 14 years and above, who had been diagnosed with complicated appendicitis and had complete medical records. Patients below 14 years, those with uncomplicated appendicitis, those with incomplete or missing medical records, and those with underlying medical conditions or comorbidities that could impact study outcomes, such as chronic renal disease or liver cirrhosis, were excluded from the study.

Data collection method

The data were mined retrospectively from patients’ medical files, following the inclusion and exclusion criteria outlined above. The extracted data included gender, age, body mass index (BMI), type of surgery, preoperative and postoperative complications, Na, potassium (K), white blood cell (WBC) count, absolute neutrophil count, neutrophil percentage, hemoglobin (Hb), and chloride.

Complicated appendicitis was defined based on the presence of intraoperative and postoperative findings, such as perforation, abscess formation, or inflammatory mass, as documented in the hospital’s electronic medical records. The classification was supported by imaging, intraoperative observations, and histopathological confirmation where available.

Ethical consideration

Ethical approval for this study was obtained from the Buraidah Central Hospital and the Regional Research Ethics Committee, which is registered with the National Committee of Bioethics (NCBE) under Registration No. H-04-Q-001 (Approval No. 607-46-1657). All study procedures complied with institutional and national ethical guidelines for research involving human participants. Data privacy and confidentiality were strictly maintained, with anonymization achieved through systematic recoding to remove all personal identifiers.

Data preprocessing and analysis

Medical records were extracted into four separate Excel files, which were merged into a single dataset using the Pandas library. As the data were not originally computerized but mined from physical medical files, standardization of entries was necessary. This was achieved using the RE:Library to harmonize text-based variables before merging all DataFrames into a unified, standardized dataset.

The outcome variable was recoded to create a new column labeled “complicated appendicitis,” derived from postoperative complications. Cases with postoperative complications were classified as complicated appendicitis, while all others were categorized as uncomplicated. The dataset had less than 5% missing data, which were handled through median imputation due to its robustness against outliers and ability to preserve data variability.

A new predictor variable, hyponatremia, was generated based on serum Na levels: values <135 mmol/L were classified as hyponatremia, 135-145 mmol/L as normonatremia, and >145 mmol/L as hypernatremia. The prevalence of complicated appendicitis and preoperative complications was then computed using Pandas.

Data analysis was conducted using Python (version 3.12.3) within the JupyterLab environment. The Pandas library was used for data cleaning, transformation, and descriptive statistics, while SciPy was applied for inferential analyses. A chi-square test of independence examined the association between complicated appendicitis and preoperative complications, assessing the relationship between preoperative and postoperative outcomes.

The outcome variable was complicated appendicitis, and the predictor variables included gender, age, BMI, type of surgery, serum Na, serum K, WBC count, and Hb. Statistical significance was set at p < 0.05.

A multivariable logistic regression analysis was performed using the statsmodels.api library with the maximum likelihood estimation method to identify independent predictors of complicated appendicitis. Both unweighted and weighted models were fitted. The weighted models incorporated stabilized inverse probability of treatment weights (IPTW) derived from propensity scores estimated using scikit-learn. Covariates in the propensity score model included BMI, WBCs, Hb, Na, K, and age. Stratified analyses were performed by sex and surgical procedure (open versus laparoscopic appendectomy) to assess subgroup-specific associations.

To enhance the robustness of results, sensitivity analyses were conducted at both the data and model levels. At the data level, 5th and 95th percentile winsorization was applied to continuous variables using pandas, numpy, and scipy.stats.mstats.winsorize to minimize the influence of extreme outliers and stabilize variable distributions. Post-winsorization comparisons of mean, standard deviation, and range confirmed that the central tendency remained stable while dispersion decreased, indicating improved dataset robustness.

At the model level, unweighted and propensity-weighted logistic regression models were compared to evaluate the stability of regression coefficients and the direction of associations under different analytical assumptions. Consistent findings across both models provided evidence of robustness in the estimated effects.

Effect sizes were assessed using Cramér’s V (via scipy.stats), while risk ratios were computed using the statsmodels.stats.api module. These complementary analyses collectively enhanced the validity, precision, and interpretability of the study findings.

## Results

Descriptive statistics

The descriptive statistics of the categorical variables in the analysis are shown in Table 1. Most of the patients were male (73.5%), the most common diagnostic procedure was CT (89.4%), and the surgical procedure was laparoscopy (64.8%). Patients with pre-existing complications accounted for 18.0%, while complicated appendicitis was reported in 5.7% of patients.

**Table 1:** Frequency distribution and proportions of demographic and clinical variables (n=878) CT, computed tomography; US, ultrasound; MRI, magnetic resonance imaging; NaN, missing data

Variable	Characteristics	Frequency	Percentage
Gender	Male	643	73.5
	Female	233	26.5
Diagnostic procedure	CT	785	89.4
	US	43	4.9
	MRI	1	0.1
	NaN	49	5.6
Surgical procedure	Laparoscopy	569	64.8
	Open	309	35.2
Preoperative complications	No	720	82.0
	Yes	158	18.0
Complicated appendicitis	No	828	94.3
	Yes	50	5.7

Table 2 summarizes the descriptive statistics of key clinical and laboratory parameters based on the winsorized data for the overall study population across age groups (pediatric and adult). The mean age for the pediatric group was 15.66 years compared to 30.76 years in adults. The pediatric group had a slightly lower BMI (23.54 kg/m²) compared to adults (26.17 kg/m²). The pediatric group also showed slightly higher WBC count values (13.66 × 10⁹/L) than adults (12.95 × 10⁹/L). The mean Hb level was slightly higher in adults (14.47 g/dL) than in pediatric patients (14.01 g/dL). Similarly, the mean serum Na level was slightly higher in adults (137.56 mmol/L) compared to pediatric patients (137.51 mmol/L). However, the mean serum K level was higher in pediatric patients (3.88 mmol/L) than in adult patients (3.79 mmol/L). For more information on the overall population, including standard deviations and ranges, see Table 2.

**Table 2:** Descriptive statistics of clinical and laboratory parameters The values in bold indicate subgroup sample sizes, while the figures in parentheses represent the ranges. BMI, body mass index; WBCs, white blood cells

Unit (SI/Clinical)	Overall (n = 878)	Pediatric patients (n = 93)	Adults (n = 785)
Age (years)	29.16 ± 10.80 (13–80)	15.66 ± 1.16 (13–17)	30.76 ± 10.31 (18–80)
BMI (kg/m²)	25.89 ± 4.64 (13.11–49.90)	23.54 ± 5.05 (15.42–43.25)	26.17 ± 4.52 (13.11–49.90)
WBCs (×10⁹/L)	13.02 ± 3.70 (6.28–20.20)	13.66 ± 3.50 (6.28–20.20)	12.95 ± 3.72 (6.28–20.20)
Hemoglobin (g/dL)	14.42 ± 1.54 (11.20–16.70)	14.01 ± 1.50 (11.20–16.70)	14.47 ± 1.54 (11.20–16.70)
Sodium (mmol/L)	137.55 ± 2.27 (133.30–142.00)	137.51 ± 2.15 (133.30–142.00)	137.56 ± 2.29 (133.30–142.00)
Potassium (mmol/L)	3.80 ± 0.30 (3.30–4.40)	3.88 ± 0.31 (3.30–4.40)	3.79 ± 0.30 (3.30–4.40)

**Figure 1:**
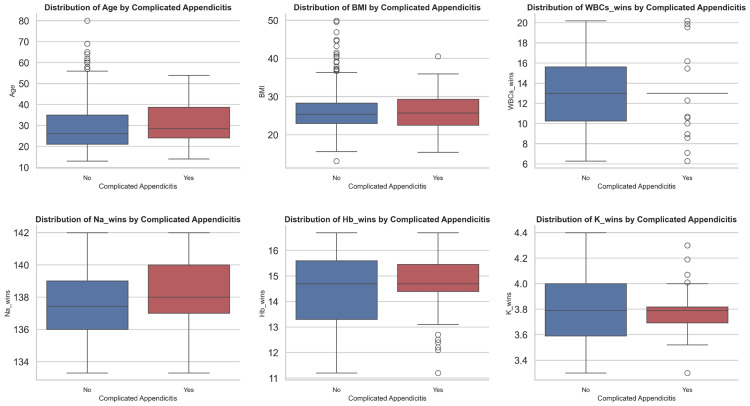
Boxplots of age, BMI, WBCs, Na, Hb, and K showing distribution and summary statistics BMI, body mass index, WBC, white blood cell, Na, sodium; Hb, hemoglobin; K, potassium

The boxplots shown in Figure 1 illustrate the distribution of key clinical and demographic variables with numerical features. Patients with complicated appendicitis tended to be older, with a higher median age compared to those with uncomplicated appendicitis. The median BMI values were comparable between the two groups, although the uncomplicated group exhibited more outliers. An elevated inflammatory response was indicated by the higher median WBC count among patients with complicated appendicitis. Hb levels were generally similar between the two groups, though patients in the uncomplicated group displayed wider variability. The median serum Na levels were lower in the uncomplicated group compared to the complicated group. Similarly, median serum K levels were marginally lower between the groups, but with greater variability observed in the uncomplicated group.

**Figure 2:**
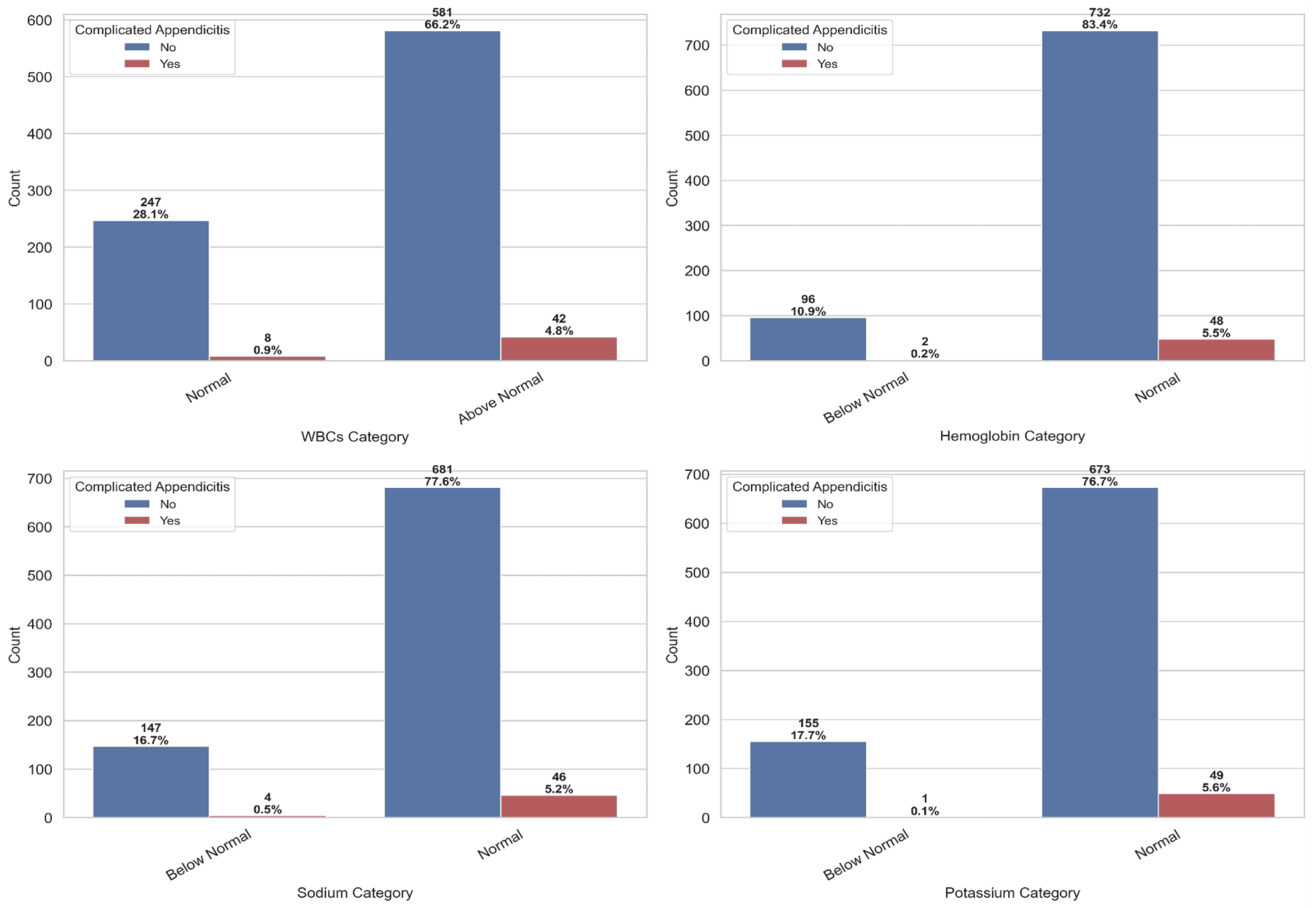
Distribution of medical parameters included in the analysis

Figure 2 presents the distribution of the medical parameters analyzed in the study. Many patients with complicated appendicitis had WBC levels above the normal range, reflecting the expected association between leukocytosis and severe infection. For Hb, most patients were within the normal range, with only a few cases falling below normal in both groups. A small number of patients with complicated appendicitis had low Na levels, while K levels remained largely normal in both groups.

**Figure 3:**
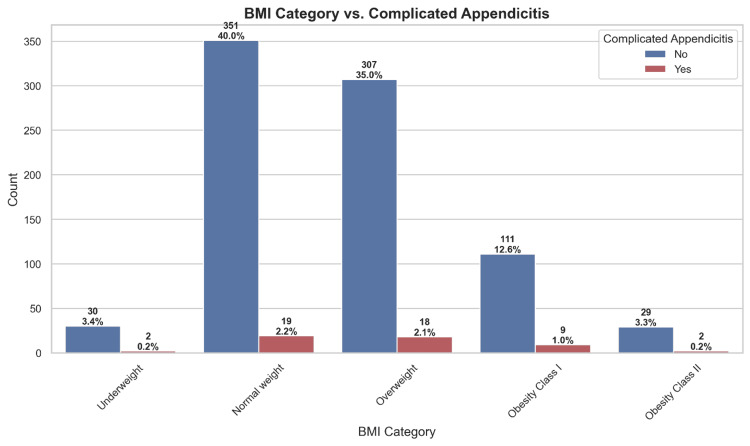
Distribution of complicated appendicitis across BMI categories BMI, body mass index

Figure 3 shows the distribution of complicated appendicitis across BMI categories. Regardless of disease severity, most patients fell within the normal weight and overweight categories, with only a small proportion of complicated cases observed among underweight and obese patients. This distribution suggests that BMI may not be a strong predictor of complications in this study population.

Sensitivity analysis

A 5th and 95th percentile winsorization was applied because the original data exhibited some visible outliers, although the team maintained confidence in most of the dataset. This procedure was intended to reduce the influence of extreme values, stabilize the variance and mean, and help determine whether the results were robust or driven by outliers. The sensitivity analysis shown in Table 3 indicates that outlier influence was present in Hb, Na, and K. The table shows that the central tendency (means) remained stable before and after winsorization, with reduced dispersion (SD and range), indicating improved robustness. Stratifying age into pediatric (≤18 years) and adult (≥18 years) groups showed no distortion between the groups. In summary, the findings of the sensitivity analysis demonstrated that the winsorized data were robust, improving precision and stabilizing the distribution of the clinical variables under investigation.

**Table 3:** Impact of winsorization on central tendency and dispersion of clinical variables BMI, body mass index, WBC, white blood cell

Unit (SI/Clinical)	Original Mean ± SD (Range)	Winsorized Mean ± SD (Range)	Change/Interpretation
Age (years)	29.16 ± 10.80 (13–80)	29.16 ± 10.80 (13–80)	No change: confirms no age outliers were capped
BMI (kg/m²)	25.89 ± 4.64 (13.11–49.90)	25.89 ± 4.64 (13.11–49.90)	No change: outliers likely already mild
WBCs (×10⁹/L)	13.08 ± 4.04 (2.50–35.89)	13.02 ± 3.70 (6.28–20.20)	Noticeable SD reduction (4.04 → 3.70); extreme highs truncated
Hemoglobin (g/dL)	14.38 ± 1.77 (5.43–18.10)	14.42 ± 1.54 (11.20–16.70)	SD drop: extreme low values removed
Sodium (mmol/L)	137.53 ± 2.84 (126–173.10)	137.55 ± 2.27 (133.30–142.00)	Big range shrink: major outliers capped
Potassium (mmol/L)	3.81 ± 0.41 (2.74–10.70)	3.80 ± 0.30 (3.30–4.40)	Very large outliers removed (10.70 → 4.40)

The winsorization procedure was guided by established clinical reference ranges for standard biochemical parameters. Normal WBC counts range from 4,500 to 11,000 cells/µL, with values below 4,500 indicating potential leukopenia and those above 11,000 suggesting leukocytosis [18]. For Hb, the normal range is 13.8-17.2 g/dL in adult males and 12.1-15.1 g/dL in adult females. Serum sodium levels typically range between 135 and 145 mEq/L, with values below 135 mEq/L indicating hyponatremia and those above 145 mEq/L suggesting hypernatremia [19]. Potassium levels normally fall between 3.5 and 5.5 mEq/L, where values below 3.5 mEq/L indicate hypokalemia and those above 5.5 mEq/L indicate hyperkalemia [20].

Chi-square association testing

The association testing results, as shown in Table 4, indicate that only the surgical procedure and diagnostic procedures had a statistically significant association with complicated appendicitis. Overall, the sample size analyzed in the present study was 878. However, the sample size for the diagnostic procedure was 829 because 49 patients had missing records.

**Table 4:** Chi-square analysis of variables associated with complicated appendicitis CT, computed tomography; US, ultrasound; MRI, magnetic resonance imaging

Variable	Characteristics	Complicated Appendicitis		p-Value
		No	Yes	
Gender (n=878)	Female	221 (25.2%)	12 (1.4%)	0.800
	Male	605 (69.1%)	38 (4.3%)	
Diagnostic procedure (n=829)	CT	758 (91.4%)	27 (3.3%)	<0.001
	MRI	1 (0.1%)	0 (0.0%)	
	US	31 (3.7%)	12 (1.4%)	
Surgical procedure (n=878)	Laparoscopy	552 (62.9%)	17 (1.9%)	<0.001
	Open	276 (31.4%)	33 (3.8%)	
Preoperative complications (n=878)	No	676 (77.0%)	44 (5.0%)	0.344
	Yes	152 (17.3%)	6 (0.7%)	

Logistic regression

The unweighted model in Table 5 presents the results from a traditional multivariable logistic regression adjusted for key biochemical and demographic covariates. This analysis aimed to estimate the covariate-adjusted association between biochemical and demographic predictors and complicated appendicitis. The biochemical and demographic predictors of complicated appendicitis revealed significant findings for Na (adjusted OR [AdjOR] = 1.180; 95% CI: 1.034-1.347) and age (AdjOR = 1.029; 95% CI: 1.004-1.055). In the female model, Hb showed a significant association (AdjOR = 2.224; 95% CI: 1.247-3.968). For males, the findings for Na (AdjOR = 1.157; 95% CI: 0.993-1.349) and age (AdjOR = 1.030; 95% CI: 0.999-1.062) were at the borderline of statistical significance. The model for open surgery revealed significant associations for Na (AdjOR = 1.222; 95% CI: 1.036-1.443) and age (AdjOR = 1.052; 95% CI: 1.015-1.092). No significant relationships were observed for laparoscopy.

**Table 5:** Multivariable logistic regression analysis of biochemical and demographic predictors of complicated appendicitis (overall and stratified by sex and surgical procedure) Notes: Unweighted Model — Traditional multivariable logistic regression adjusted for biochemical and demographic covariates, without weighting. Weighted Model  — Weighted logistic regression using stabilized IPTW derived from propensity scores, trimmed at the 5th and 95th percentiles, to estimate marginal effects. Covariates included in the propensity score model were BMI, WBCs, Hb, serum N), serum K, and age. Figures in bold represent p-values, while the rest represent odds ratios (OR).**Denotes statistical significance at the p < 0.05 level. IPTW, inverse probability of treatment weights; BMI, body mass index, WBC, white blood cell, Hb, hemoglobin; Na, sodium; K, potassium; N/A, not applicable

Predictors	Unweighted (Model 1)	Females	Males	Propensity Weighted (Model 1)	Open Surgery	Laparoscopy	Propensity Weighted (Model 2)
(1) BMI	0.990	1.054	0.968	0.994	0.969	1.016	1.004
	0.758	0.389	0.443	0.870	0.533	0.402	0.907
(2) WBCs	0.999	0.996	0.999	1.015	0.995	0.997	0.999
	0.974	0.969	0.986	0.707	0.930	0.962	0.992
(3) Hb	1.097	2.224**	0.891	0.988	0.977	1.092	1.035
	0.380	0.007	0.452	0.918	0.881	0.599	0.740
(4) Na	1.180**	1.289	1.157	1.207**	1.222**	1.089	1.168**
	0.014	0.074	0.062	0.007	0.018	0.453	0.021
(5) K	0.762	1.172	0.611	0.795	0.592	0.843	0.731
	0.589	0.894	0.394	0.659	0.433	0.838	0.536
(6) Age	1.029**	1.005	1.030	1.027**	1.052**	1.016	1.029**
	0.025	0.855	0.055	0.041	0.006	0.447	0.021
Constant	0.000	0.000	0.020	0.000	0.000	0.000	0.000
	0.004	0.012	0.074	0.003	0.015	0.269	0.008
Observations	878	233	645	878	309	569	878
Log-likelihood	-185.55	-39.43	-140.41	-167.86	-98.64	-75.11	-183.04
LL-Null	-191.83	-47.28	-144.46	N/A	-104.99	-76.43	N/A
LLR p-value	0.051	0.015	0.231	N/A	0.048	0.853	N/A

Propensity-weighted logistic regression

The weighted model in Table 5 also includes results from a propensity-weighted logistic regression using stabilized IPTW. These weights were derived from a propensity score model that included age, BMI, WBCs, Hb, serum Na, and serum K levels. To minimize the influence of extreme weights, we trimmed them at the 5th and 95th percentiles. This approach helped account for measured confounding by weighting patients to mimic a randomized distribution of the exposures: gender and surgical procedure.

There was an initial gender imbalance (male = 645, female = 233). After applying IPTW, the weighted multivariable logistic regression model remained statistically significant overall (LLR p = 0.051, pseudo R² = 0.013). Age (β = 0.027, p = 0.041) and serum Na (β = 0.188, p = 0.007) showed significant positive associations with complicated appendicitis.

Surgical procedure was categorized as open surgery (n = 309) and laparoscopy (n = 569). Similarly, after weighting, the model remained statistically significant (LLR p = 0.048, Pseudo R² = 0.013). Age (β = 0.029, p = 0.021) and serum Na (β = 0.155, p = 0.021) were again significantly associated with the outcome.

BMI, WBCs, Hb, and K were not statistically significant after rebalancing. A comparison between the weighted and unweighted models indicated that the coefficients remained stable, supporting the robustness of the logistic regression results. This suggests that the initial imbalance in gender and surgical procedure did not materially affect the observed associations.

## Discussion

Hyponatremia as a marker of complicated appendicitis

This paper reviews hyponatremia as a predictor of complicated appendicitis among patients at Buraidah Central Hospital.

The overall prevalence of hyponatremia in the study population was 11.3% (99/878; 95% CI: 9.35-13.54). Among patients with complicated appendicitis, the prevalence of hyponatremia was 4.0% (4/100; 95% CI: 1.10-13.46). When stratified by age, hyponatremia was observed in 6.5% of pediatric patients (6/93; 95% CI: 2.99-13.37) and 11.8% of adult patients (93/785; 95% CI: 9.77-14.30). The relatively small number of pediatric cases may have limited the statistical power of subgroup analyses.

The overall prevalence of complicated appendicitis was 5.69% (95% CI: 4.35-7.43). When the data were stratified by age group and gender, some variations were observed. Among adult females, the prevalence was 5.94% (12/202; 95% CI: 3.43-10.10), while adult males had a slightly higher prevalence of 6.17% (36/583; 95% CI: 4.49-8.43). In contrast, the prevalence was lower among the pediatric group. There were no cases of complicated appendicitis among pediatric females (0.0%, 0/31; 95% CI: 0.00-11.03), whereas pediatric males had a prevalence of 3.23% (2/62; 95% CI: 0.89-11.02). Overall, these findings suggest that complicated appendicitis is more common in adults than in children, with male patients - both adult and pediatric - showing slightly higher prevalence rates compared to females. However, the wider confidence intervals in the pediatric cohort reflect smaller sample sizes and greater statistical uncertainty in these estimates. The differences in these prevalence figures were non-significant, an indication that gender would not be a strong independent risk factor for developing complicated appendicitis. A similar study by Alotaibi et al. [21] in Saudi Arabia reported the prevalence of complicated appendicitis as 13.4%, while the study by Alfayez et al. [22] at Prince Sultan Military Medical City reported 19%, both of which were higher compared with the present study.

The overall prevalence of preoperative complications was 18.15% (95% CI: 15.60-20.67). When stratified by age, the prevalence among adults was 18.7% (147/785; 95% CI: 16.15-21.61) compared to 11.8% (11/93; 95% CI: 6.73-19.95) in pediatric patients. Preoperative complications were significantly more common among patients with hyponatremia, particularly in adults, where the prevalence was 59.1% (55/93; 95% CI: 48.98-68.58) compared to 13.3% (92/692; 95% CI: 10.97-16.03) in those without hyponatremia. Among pediatric patients, a similar trend was observed: 33.3% (2/6; 95% CI: 9.68-70.00) in those with hyponatremia versus 10.3% (9/87; 95% CI: 5.54-18.51) in those without. However, the wide confidence intervals in this group indicate considerable uncertainty due to the small sample size.

The prevalence of preoperative complications was higher among women at 22.3% (52/233; 95% CI: 17.45-28.09) compared to 16.4% (106/645; 95% CI: 13.78-19.49) in men. However, the presence of hyponatremia significantly increased complication rates in both genders, rising to 53.8% (21/39; 95% CI: 38.57-68.43) in women and 60.0% (36/60; 95% CI: 47.37-71.43) in men. In contrast, among patients without hyponatremia, the rates were much lower at 16.0% (31/194; 95% CI: 11.49-21.79) for women and 12.0% (70/585; 95% CI: 9.58-14.85) for men. These findings underscore hyponatremia as a strong predictor of adverse preoperative outcomes across both sexes. Although the differences were non-significant, the finding could be indicative of gender-based disparities in the presentation of acute appendicitis, delays in diagnosis, or differences in the physiological body responses to acute inflammation.

The mean WBC level in the cohort was 13.02 ± 3.70 × 10⁹/L. Leukocytosis (>11 × 10⁹/L) was present in 84.0% (42/50) of patients with complicated appendicitis compared to 70.2% (581/828) of those with uncomplicated appendicitis. This difference was statistically significant (Z = 2.09; p = 0.036). The chi-square test showed a borderline association between leukocytosis and complicated appendicitis (χ² = 3.73; p = 0.053). No significant difference in mean WBC levels was observed between the two groups (p = 0.663). Overall, these findings highlight the clinical relevance of leukocytosis as an inflammatory marker associated with disease severity in acute appendicitis. A study by Eddama et al. [23] showed that elevated levels of WBCs were a predictor of complicated appendicitis.

The study also investigated the relationship between hyponatremia and complicated appendicitis using the standard clinical threshold of serum Na concentration <135 mEq/L. A confusion matrix was constructed to compare predicted hyponatremia status with actual complication status. Of the total 878 patients, 731 uncomplicated appendicitis cases were correctly identified as not hyponatremic (true negatives), 97 were incorrectly identified as hyponatremic (false positives), 48 complicated cases were incorrectly identified as not hyponatremic (false negatives), and only two complicated cases were correctly identified as hyponatremic (true positives).

Overall, 11.3% of patients had hyponatremia, and 5.69% had complicated appendicitis. The sensitivity (true positive rate) was 4.0%, indicating that hyponatremia detected only a small fraction of complicated cases. In contrast, the specificity (true negative rate) was 88.3%, showing that the absence of hyponatremia strongly aligns with uncomplicated cases. These results suggest that hyponatremia alone is not a reliable standalone predictor of complicated appendicitis, though its high specificity supports its role as a potential “rule-out” marker when used in combination with other clinical and laboratory findings. This aligns with evidence from Anand et al. [17], Pham et al. [24], and Walsh et al. [25], which suggests that hyponatremia may play a supportive but not definitive diagnostic role.

A low Hb value of 5.43 g/dL was reported prior to winsorization, which could be associated with a chronic disease or bleeding. The mean Hb level in the present study was 14.42 ± 1.54 g/dL, with values ranging from 11.20 to 16.70 g/dL. Hb levels were largely within the normal range in both groups, with mean levels of 14.40 g/dL among patients with uncomplicated appendicitis and 14.66 g/dL among those with complicated appendicitis. Statistical analysis showed no significant difference in Hb levels between the two groups (t = -1.460; p = 0.150). These findings suggest that anemia was not a distinguishing feature between complicated and uncomplicated appendicitis in this cohort. While Hb may not directly predict appendicitis complications, it could still play a role in influencing post-operative recovery.

K and Na levels were generally normal in both groups. The mean Na level was 137.55 ± 2.27 mEq/L (range 133.30-142.00), and the mean K level was 3.80 ± 0.30 mEq/L (range 3.30-4.40). Na was significantly higher in complicated cases (138.37 mEq/L, 95% CI 137.76-138.99) compared to uncomplicated cases (137.50 mEq/L, 95% CI 137.35-137.66), with t = 2.749, p = 0.0080. K did not differ significantly (3.78 vs 3.80 mEq/L, 95% CIs 3.73-3.83 and 3.78-3.82; t = -0.718, p = 0.4754). Hyponatremia prevalence was 4.00% (95% CI 1.10-13.46) in complicated cases versus 11.71% (95% CI 9.70-14.08) in uncomplicated cases (p = 0.0939, NS), while hypokalemia was 2.00% (95% CI 0.35-10.50) versus 14.98% (95% CI 12.71-17.57), p = 0.011. Although statistically significant differences were observed for Na and hypokalemia, absolute differences were small and values remained within normal physiological ranges. These findings highlight that electrolyte disturbances are not strong diagnostic markers of complicated appendicitis but may still hold clinical relevance for perioperative care. Statistical significance should be interpreted in the clinical context, not in isolation.

Clinical and procedural factors associated with complicated appendicitis

The present findings showed that diagnostic procedure (χ² = 72.61, p < 0.001) and surgical procedure (χ² = 20.65, p < 0.001) were significantly associated with complicated appendicitis. The strength of association was moderate for diagnostic procedure (Cramér’s V = 0.29) and small-to-moderate for surgical procedure (Cramér’s V = 0.15). In contrast, there was insufficient evidence to link gender (p = 0.800) or preoperative complications (p = 0.344) with complicated appendicitis, with both showing very weak or negligible effect sizes (Cramér’s V < 0.05).

The male patients showed a slightly higher percentage of complicated appendicitis (5.9%; 95% CI: 4.3-8.0) compared to females (5.2%; 95% CI: 3.0-8.8). However, this difference was not statistically significant (Z = 0.418, p = 0.676). These findings align with a prior report by Kollias et al. [26], which observed a higher incidence in males. In this study, however, the evidence was insufficient to establish a gender-based risk. Given the skewed sex distribution (645 males [73.5%] vs 233 females [26.5%]) and the retrospective, single-center design, the non-significant result should be interpreted with caution.

The choice of diagnostic imaging modality was significantly associated with complicated appendicitis (χ²(2) = 54.49, p < 0.001; Cramér’s V = 0.26). Among patients who underwent CT scans, 758 (96.6%) had uncomplicated appendicitis, while 27 (3.4%; 95% CI: 2.4-5.0) had complicated disease. In contrast, 12 (27.9%; 95% CI: 16.5-42.7) of the 43 patients who underwent ultrasound were in the complicated group, indicating a higher proportional use of ultrasound among complicated cases. MRI was rarely used (n = 1), with a wide confidence interval reflecting the limited sample. These findings are consistent with previous studies by Lu et al. [27] and Raffa et al. [28], which reported that CT scans have a higher detection rate of complicated appendicitis compared to ultrasound. The present findings suggest that CT remains the preferred modality for timely and accurate diagnosis, potentially preventing progression to complicated appendicitis.

The surgical techniques used in the management of appendicitis showed statistically significant differences between patients with uncomplicated and complicated disease (χ² = 20.65, df = 1, p < 0.001). Among patients with uncomplicated appendicitis, laparoscopic appendectomy was performed in 66.7% (552/828; 95% CI: 63.4%-69.8%) of cases, while open surgery accounted for 33.3% (276/828; 95% CI: 30.2%-36.6%). In contrast, among patients with complicated appendicitis, open surgery was used more frequently in 66.0% (33/50; 95% CI: 52.2%-77.6%) of cases compared with 34.0% (17/50; 95% CI: 22.4%-47.8%) for laparoscopic surgery. These findings indicate a significant association between surgical technique and disease severity, with laparoscopy being more common in uncomplicated cases, whereas open surgery predominated in complicated cases.

These findings are consistent with previous studies by Biondi et al. [29], Jailani et al. [30], and Alotaibi et al. [21], which reported that laparoscopic appendectomy was predominantly performed in uncomplicated cases, whereas open appendectomy was more frequently employed in complicated cases. This supports the notion that disease severity significantly influences the choice of surgical technique.

Among patients without complicated appendicitis, no preoperative complications were observed in 81.6% (676/828; 95% CI: 78.9%-84.1%), while preoperative complications occurred in 18.4% (152/828; 95% CI: 15.9%-21.1%). In contrast, among patients with complicated appendicitis, no preoperative complications were observed in 88.0% (44/50; 95% CI: 76.2%-94.4%) and preoperative complications in 12.0% (6/50; 95% CI: 5.6%-23.8%). The association between preoperative complications and complicated appendicitis was not statistically significant (χ²(1) = 0.897, p = 0.344), and the effect size was negligible (Cramér’s V = 0.032), indicating a very weak relationship. These findings suggest that pre-existing appendicitis conditions may not directly influence progression to complicated appendicitis, though the limited sample size warrants cautious interpretation.

Biochemical and demographic predictors of complicated appendicitis

The multivariable logistic regression analysis of biochemical and demographic predictors of complicated appendicitis identified serum Na and age as the key significant predictors in the overall unweighted and propensity-weighted models.

In the unweighted model, higher serum Na levels were significantly associated with increased odds of complicated appendicitis (OR = 1.180, p = 0.014). This association remained significant after applying propensity weighting, confirming the robustness of Na as a predictor even after adjusting for confounders. In the gender-weighted model (model 1), each 1 mEq/L increase in Na corresponded to a 20.7% increase in the odds of complicated appendicitis (OR = 1.207, p = 0.007), while in the surgery-weighted model (model 2), the odds increased by 16.8% (OR = 1.168, p = 0.021). In contrast to the initial hypothesis and previous studies [11-17], which consistently associated hyponatremia (low Na) with increased disease severity among both adult and pediatric patients, the present study identified a positive association between higher serum Na levels and complicated appendicitis. This paradoxical finding suggests that the relationship between serum Na and disease complexity may vary across populations and clinical contexts, warranting cautious interpretation and further investigation.

Age was also a consistent predictor, with each additional year associated with a 2.9% increase in the odds of complicated appendicitis (unweighted OR = 1.029, p = 0.025; weighted model 1 OR = 1.027, p = 0.041; weighted model 2 OR = 1.029, p = 0.021). These findings are consistent with previous studies, including those by Eddama et al. [23] and Alotaibi et al. [21], which reported that increased age is a strong predictor of complicated appendicitis.

Although Hb was not significant in the overall model (OR = 1.097, p = 0.380), stratified analysis by sex showed that it was significantly associated with complicated appendicitis among females (OR = 2.224, p = 0.007), suggesting a possible gender-related physiological or clinical difference. However, this effect did not persist after propensity weighting (OR = 0.988, p = 0.918), indicating that the initial association may have been partly due to confounding factors.

WBCs, K, and BMI were not significantly associated with complicated appendicitis in either unweighted or weighted models (p > 0.05 across all strata). This contrasts with some prior literature that suggested elevated WBCs may be a biomarker for complicated appendicitis; for example, Alfayez et al. [22] observed such an association in their cohort. The lack of significance in our study may reflect population differences, clinical practice variations, or confounding by other biochemical or demographic factors.

Stratification by surgical procedure revealed that Na (OR = 1.222, p = 0.018) and age (OR = 1.052, p = 0.006) remained significant predictors among patients undergoing open surgery, whereas no predictor reached statistical significance among those who underwent laparoscopy. This suggests that older patients and those with higher Na levels are more likely to present with complicated appendicitis in cases managed surgically by open appendectomy. These findings mirror trends observed in prior studies where age was associated with more advanced disease at presentation [21,23].

Across both the chi-square test of association and the risk ratio analysis, hyponatremia was not significantly associated with complicated appendicitis. Although the proportion of hyponatremia was lower among patients with complicated appendicitis (4.0%, 95% CI: 1.1%-13.5%) compared to those without (11.7%, 95% CI: 9.7%-14.1%), this difference was not statistically significant based on the Z-test for proportions (p = 0.094) and the chi-square test (p = 0.149), with a negligible effect size (Cramér’s V = 0.049). Similarly, patients with hyponatremia had a 2.0% risk of complicated appendicitis, compared to 6.2% among those without hyponatremia. Although the risk ratio was 0.33 (95% CI: 0.08-1.33), the confidence interval included 1, meaning the observed difference was not statistically significant. Taken together, these findings reinforce that hyponatremia alone was not a strong predictor of complicated appendicitis, consistent with prior evidence questioning its standalone predictive utility.

Study limitations

This study’s retrospective design and reliance on medical records from 2020 to 2023 introduced potential risks of data entry errors and reporting bias, although procedures such as winsorization were applied to reduce skewness. Future investigations could benefit from integrating electronic health record systems that automatically update from digitized databases to enhance data accuracy and completeness.

Although a census sampling approach was used to include all eligible patients treated for complicated appendicitis during the study period, the use of data from a single hospital may limit the generalizability of the findings. The reliance on retrospectively collected records of patients presenting to the emergency department also means that cases managed in other facilities or within the wider regional population may not have been captured. Nonetheless, by including all consecutive eligible cases over a four-year period, potential selection bias was minimized, thereby improving the representativeness of patients treated at this center.

With an overall prevalence of 11.85% in adults and 6.45% in children, the combined prevalence of complicated appendicitis with hyponatremia was relatively low (11.28%). This comparatively low occurrence may have reduced the statistical power of some analyses and limited the generalizability of the results to populations with a higher disease burden. It also suggests that the study cohort may not fully reflect broader epidemiological trends of hyponatremia in complicated appendicitis, particularly in low-resource settings. These factors should therefore be considered when interpreting the findings.

Another limitation of this study is that several biochemical variables, such as K, chloride, absolute neutrophil count, and neutrophil percentage, were initially extracted during data collection but later excluded from analysis due to substantial missing data. These parameters are biologically linked to both electrolyte balance and the inflammatory response, and their exclusion may have introduced residual confounding. Consequently, the observed association between serum Na and complicated appendicitis should be interpreted with appropriate caution.

The inclusion of only patients with complicated appendicitis introduced a potential spectrum bias, as uncomplicated cases were excluded by design (see inclusion and exclusion criteria). This selective inclusion limited the study’s ability to fully evaluate the predictive performance of hyponatremia across the entire spectrum of appendicitis severity.

Practical implications

For the multivariate analysis, additional medical parameters can be added in the model to improve the predictive accuracy. For future studies, it would be important to investigate the combined markers, for example, the use of hyponatremia with hyperbilirubinemia to test if it would enhance the diagnostic performance. A future prospective study would be necessary to validate the findings and assess the use of hyponatremia as a predictor. Future studies can also focus on testing machine learning algorithms, specifically reinforcement learning, which can be used to develop and deploy an application that can help improve the outcomes of surgical interventions in the management of complicated appendicitis.

## Conclusions

In conclusion, this study highlights that serum Na levels and patient age stand out as the most consistent and meaningful predictors of complicated appendicitis in this cohort. Propensity score analysis using IPTW further reinforced the robustness of these predictors. In contrast, WBC count, K, BMI, and Hb did not show significant independent associations after adjustment, suggesting that their role in predicting disease severity may be limited.

Notably, contrary to the initial hypothesis and previous literature that associated hyponatremia with increased disease severity, this study found a positive association between higher serum Na levels and complicated appendicitis. This paradoxical finding warrants cautious interpretation and further investigation in larger, multicenter studies.

Although hyponatremia remains clinically relevant, it is not sufficient on its own to identify complicated cases. However, when considered together with patient age, serum Na provides meaningful support for early risk stratification and timely treatment decisions. These findings underscore the importance of integrating these simple and readily available measures into routine clinical assessments to enhance the early identification and management of patients with suspected complicated appendicitis.
